# The treatment and risk factors of retinopathy of prematurity in neonatal intensive care units

**DOI:** 10.1186/s12886-018-0973-1

**Published:** 2018-11-20

**Authors:** Yunxia Leng, Wenzhi Huang, Guoliang Ren, Cheng Cai, Qingbiao Tan, Yuqin Liang, Weizhong Yang, Zongyin Gao

**Affiliations:** 1 Guangzhou First People’s Hospital, Guangzhou city, China; 2 Second Affiliated Hospital of South China University of Technology, Guangzhou city, China

**Keywords:** Anti-VEGF, Neonatal intensive care unit/NICU, Retinopathy of prematurity/ROP

## Abstract

**Background:**

Retinopathy of prematurity (ROP) is a vascular proliferative disorder of the developing retina and a significant cause of childhood blindness around the world. The incidence of ROP is affected by many factors, and the incidence rate varies from country to country. The purpose of this study is to report the incidence and risk factors of ROP in neonatal intensive care unit (NICU) of Guangzhou First People’s Hospital in China.

**Methods:**

A retrospective review was performed on 436 premature infants who were consecutive ROP screened in the NICU of Guangzhou First People’s Hospital from March 2013 to October 2017. The single-factor analysis and the logistic multivariate regression analysis were used to detect risk factors of ROP.

**Results:**

Total 436 premature infants were consecutive ROP screened, 138 (31.65%) were found ROP, and 61(13.99%) were treated. The single-factor analysis revealed that the incidence of ROP was associated with multiple births, gestational age, birth weight, mechanical ventilation, intravascular hemolysis, the number of operations and blood culture results. The logistic multivariate regression analysis revealed that gestational age; birth weight, mechanical ventilation, minimum SaO2 and daily weight gain were independent risk factors for ROP onset. Forty-nine patients underwent retinal laser photocoagulation with recurrence 20 patients. Twelve patients underwent anti-VEGF drug (Ranibizumab) via intraocular injection with 5 patients of recurrence.

**Conclusions:**

The incidence of ROP in NICU of Guangzhou China will match those in middle-income countries, but higher than high-income countries. Anti-VEGF drugs could be preferred as a good treatment method for zone 1 ROP and aggressive posterior ROP.

## Introduction

Retinopathy of prematurity (ROP) is one of the major causes of blindness in children and is the most common cause of retinal vasculopathy in premature or low-birth-weight infants [1]. The physiological process by which the retina is vascularized in human embryos is divided into two stages: vasculogenesis and angiogenesis. Vasculogenesis is the process that occurs during the early stage of retinal vascularization in which endothelial progenitor cells differentiate into endothelial cells to form blood vessels. This process begins at 12 weeks and finishes at 21 weeks of gestational age. Angiogenesis is a process that occurs in the advanced stage of retinal vascularization, during which the superficial plexuses that are responsible for the central hemal arch form. The blood vessels gradually grow and develop to surround the retina from the optic disk beginning at 16 weeks of gestational age, reaching the nasal retina at 32 weeks and the temporal retina at 36 to 40 weeks [2–4]. However, in premature babies the retinal blood vessels it is incomplete. With many factors, the vessels maybe grow and branch abnormally, and then ROP would develop. These abnormal blood vessels may grow up from the plane of the retina and may bleed inside the eye. When the blood and abnormal vessels are reabsorbed, it may give rise to multiple bands like membranes, which can pull, up the retina, causing detachment of the retina and eventually blindness before 6 months.

In recent years, with the development of neonatal medical technology, an increasing number of severe premature infants have survived, including cases of prematurity associated with in vitro fertilization (IVF), multiple births and infants with very low birth weight, very low gestational age, congenital dysplasia, immaturity, septicemia, severe infections and multiple surgeries after birth. The survival rates of extremely immature premature infants < 26 weeks and < 1000 g worldwide have continuously increased, and the incidence of ROP has been increasing in parallel [5–8].

This study retrospectively analyzed the screening and treatment of ROP in severe premature infants who were admitted to the Neonatal Intensive Care Unit (NICU) of Guangzhou First People’s Hospital from March 2013 to October 2017. The results revealed that ROP was common in severe premature infants with risk factors and that anti-VEGF treatment of ROP produced a curative effect. Guangzhou First People’s Hospital is a comprehensive Grade 3A hospital in economically developed regions of China with advanced neonatal care. The purpose of this study was to analyze the prevalence and risk factors of ROP in advanced neonatal care hospitals in a single centre of China.

## Subjects and methods

### Ethical approval

The medical ethics committee of Guangzhou First People’s Hospital approved this study. The patient-related data collected in this study include the following: name, age, and clinical diagnosis. The names were replaced by numbers, and the relevant data of the patient was encrypted and stored in the main study. We had obtained the written informed consent of the parents of the children.

### Research subjects

A retrospective analysis was performed on premature infants who underwent ROP screening in the NICU of Guangzhou First People’s Hospital from March 2013 to October 2017.

### Screening criteria

According to the 2004 and 2014 China ROP screening guidelines [9], ROP screening was performed on premature and low birth weight infants with a gestational age < 32 weeks or birth weight < 2000 g. For infants with severe illness or a clear history of oxygen intake over a long period of time, ROP screening standards were appropriately relaxed.

### Screening methods

The first fundus examination was performed at 4 to 6 weeks after birth or at 32 weeks of postmenstrual age (PMA). The following methods and procedures were used: (1) the primary screening was performed by binocular indirect ophthalmoscopy with appropriate mydriasis. Infants with suspected ROP were examined with Retcam (retina camera). (2) The surgeon determined the use and method of treatment based on the results and images got from the Retcam examination. (3) Examination results and treatment recommendations were recorded in detailed electronic medical records. They are location of the disease into zones (1, 2, and 3), the circumferential extent of the disease based on the clock hours (1–12), the severity of the disease (stage 1–5) and the presence or absence of “Plus Disease”. And screen or treatment information, which included follow-up, treatment methods. (4) Follow-up treatments and condition progression were recorded in detailed electronic medical records according to the international staging and classification standard for ROP [10]. They are location of the disease into zones (1, 2, and 3), the circumferential extent of the disease based on the clock hours (1–12), the severity of the disease (stage 1–5) and the presence or absence of “Plus Disease”.

### Treatment methods

Laser photocoagulation and anti-VEGF drug (Ranibizumab) were used in 61 premature infants with ROP who reached the therapeutic threshold. Forty-nine with ROP in zone 2 or zone 3 underwent retinal laser photocoagulation, and 12 with ROP in zone 1 or aggressive posterior ROP (AP-ROP) were given anti-VEGF drug (Ranibizumab 0.25 mg) via intraocular injections. All patients with ROP recurrence were retreated with laser photocoagulation. Infants with ROP were monitored until 54 weeks of PMA as the study endpoint.

### Data collection

Data related to the objectives were collected from the electronic medical record. Data included the following: (1) birth data (gestational age, single/multiple pregnancy, gender, body weight, head circumference, body length, etc.), (2) relevant data obtained within 30 days after birth (average daily weight gain, blood oxygen saturation, mechanical ventilation, intravascular hemolysis (IVH) and congenital dysplasia, etc.), (3) long-term follow-up data for infants with ROP (treatment regimen, number of treatments and curative effect).

### Statistical analysis

SPSS 17.0 (SPSS Inc. Chicago, IL) was used for all statistical analyses. A single-factor analysis was used to analyze correlations with ROP and identify a normal fundus (ROP (−) or ROP (+). All measurement data are presented as percentages (%) and were measured with the χ^2^ test. A multivariate logistic regression analysis was used to determine independent risk factors for ROP. *P* < 0.05 was defined as the threshold for a statistically significant difference.

## Results

### Baseline clinical information

The total number of newborns was 11,275 in Guangzhou First People’s Hospital from March 2013 to October 2017. Four hundred thirty-six severely premature infants met the ROP screening criteria and completed a first ROP ophthalmological examination at which their general condition was recorded. Among these patients, 256 (58.72%) were boys, and 180 (41.28%) were girls. The incidence of all stages ROP was 31.25% (80) girls and 32.22% (58) boys. The treatment of ROP was 12.89% (33) girls and 15.56% (28) boys. There are 286 (65.60%) were single births, 116 (26.60%) were twin births, and 34 (7.80%) were gestations births. The incidence of ROP was 33.57% (96) in single births, 23.28%(27) in twin births, and 44.12%(15) in gestations births. The treatment of ROP was 12.24%(35) in single births, 13.79%(16) in twin births, and 44.12%(29.41) in gestations births. The average gestational age was 33.84 weeks (min 26 max 39 weeks). Overall, 342 of the patients (78.44%) had a gestational age ≤ 32 weeks, and 94 patients (21.56%) had a gestation age > 32 weeks. The incidence of ROP were 31.56% (117) in gestational age < 32 weeks and 22.34% (21) in gestational age > 32 weeks. The treatment of ROP were 16.4%(56) in gestational age < 32 weeks and 5.3%(5) in gestational age > 32 weeks. The mean birth weight was 2160.23 g (min 1015 max 3690 g). Birth weights ≤1500 g were recorded in 251 cases (57.57%), weights 1501–2000 g were recorded in 109 cases (25%), and weights > 2000 g were recorded in 76 cases (17.43%). The incidence of ROP were 46.61% (117) in birth weights ≤1500 g, 16.51% (18) in birth weights 1501-2000 g, and 3.95% (3) in birth weights > 2000 g. The treatment of ROP was 21.51%(54) in birth weights ≤1500 g, 6.42% (7) in birth weights 1501-2000 g, and 0 in birth weights > 2000 g. One hundred forty-two of the patients (32.57%) were mechanical ventilation > 96 h group, and 294 patients (67.43%) were mechanical ventilation < 96 h group. The incidence of ROP were 54.22% (77) in mechanical ventilation > 96 h group, and 20.75%(61) in mechanical ventilation < 96 h group. The treatment of ROP were 28.17% (40) in mechanical ventilation > 96 h group, and 7.87%(21) in mechanical ventilation < 96 h group.

### Analysis of ROP pathogenesis

#### Single-factor correlation analysis

According to the screening results and treatment, all children were divided into two groups: ROP (−) and ROP(+). Four hundred thirty-six severely premature infants, 298 patients (68.35%) were ROP (−), and 138 patients (31.65%) were ROP (+). In ROP(+) group, 61 patients (44.20%) received treatment (12 patients revieved anti-VEGF drug treatment and 49 patients received laser treatment). The ROP (+) and ROP (−) groups were included in a univariate analysis. The results showed that the incidence of ROP was correlated with many factors including multiple birth, gestational age, birth weight, mechanical ventilation, IVH, number of operations, and positive blood culture (Table 1**)**

**Table 1:** Single factor correlation analysis

Items	No.	ROP (+)/case (%)	ROP(−) /case (%)	OR (95%CI)	χ2	P
Gender				0.956(0.635~ 1.440)	0.046	0.830
F	256	80 (31.25)	176 (68.75)			
M	180	58 (32.22)	122 (67.78)			
Number of fetuses				(not a 2 × 2 table, could not calculate)	6.689	0.035
Single birth	286	96 (33.57)	190 (66.43)			
Twin birth	116	27 (23.28)	89 (76.72)			
Multiple birth	34	15 (44.12)	19 (55.88)			
Gestational age				1.808(1.059~ 3.084)	4.802	0.028
≤ 32 w	342	117 (34.21)	225 (65.79)			
>32 w	94	21 (22.34)	73 (77.66)			
Birth weight				(not a 2 × 2 table, could not calculate)	74.550	0.000
≤ 1000 g		0	0			
~ 1500 g	251	117 (46.61)	134 (53.39)			
~ 2000 g	109	18 (16.51)	91 (83.49)			
> 2000 g	76	3 (3.95)	73 (96.05)			
Mechanical ventilation > 96 h				4.525(2.931~ 6.994)	49.605	0.000
Y	142	77 (54.22)	65 (45.78)			
N	294	61 (20.75)	233 (79.25)			
Intravascular hemolysis				3.095(2.037~ 4.701)	29.064	0.000
Y	169	79 (46.75)	90 (53.25)			
N	267	59 (22.09)	208 (77.91)			
Number of operation				(not a 2 × 2 table, could not calculate)	22.246	0.000
≥ 2	30	13 (43.33)	17 (56.67)			
1	111	54 (48.64)	57 (51.35)			
0	295	74 (25.08)	221 (74.92)			
Blood culture				2.299(1.260~ 4.194)	7.662	0.006
Positive	49	24 (48.98)	25 (51.02)			
Negative	387	114 (29.46)	273 (70.54)			

#### Multifactor nonconditional logistic regression analysis

Independent variables found to be highly related to ROP in previous publications and significantly related to ROP in the single-factor analysis of variance performed in the present study (*P* < 0.05), which were introduced into a logistic regression equation for a multi-factor unconditional logistic regression analysis. The independent risk factors that were introduced into the logistic regression model included gestational age, birth weight, mechanical ventilation, oxygen saturation, and average daily weight gain (Table 2).

**Table 2:** Multifactor non-conditional logistic regression analysis

Variables in the equation	Regression coefficient	Standard deviation	Wald	P	OR	95% CI of EXP (B)
Gestational age	−0.338	0.103	10.682	0.001	0.713	0.582–0.873
Birth weight	−0.002	0.001	6.771	0.009	0.998	0.996–0.999
Mechanical ventilation	−0.046	0.392	0.014	0.006	0.955	0.443–2.060
Minimum SaO2	−0.058	0.025	5.532	0.019	0.944	0.899–0.990
Average daily weight gain	−1.843	0.938	3.858	0.050	0.158	0.25–0.996

### Selection and effect analysis of ROP treatments

#### Selection of ROP treatments

Among the 138 included premature infants with ROP, 61 achieved the treatment threshold, including 49 patients with ROP in zone 2 or zone 3 who underwent retinal laser photocoagulation and 12 with ROP in zone 1 or AP-ROP who were given anti-VEGF drug (Ranibizumab) via intraocular injections. Ranibizumab was injected into the vitreous with 0.25 mg per dose. In the laser photocoagulation treatment group, 21 patients (42.86%) exhibited recurrence of ROP. In the Ranibizumab treatment group, 5 patients (41.67%) had recurrence of ROP. All infants with ROP recurrence were retreated with laser photocoagulation. The conditions of all infants were controlled after retreatment until 54 weeks of PMA. No serious complications such as retinal detachment occurred in any patient in our study. The results of the analysis showed that both the number of gestational weeks and birth weight were significantly lower in the Ranibizumab treatment group than in the laser photocoagulation treatment group and the untreated ROP (+) group (Table 3).

**Table 3:** Comparison of gestational age and birth weight with different treatment groups

Treatment	Cases (N)	Gestational age (weeks)	Birth weight (g)
Anti-VEGF	12	28.76 ± 3.16	1074 ± 204
Laser treatment	49	31.37 ± 2.58	1566 ± 229
Non treatment	77	33.88 ± 2.24	2272.79 ± 338
Total	138	*P* < 0.01	*P* < 0.01

## Discussion

The proportion of blindness as a result of ROP varies greatly among countries and is influenced by both the level of neonatal care and the availability of effective screening and treatment programs [11, 12]. A global perspective of the epidemiological studies of ROP showed that ROP has exhibited three epidemics since it was first described in the 1940s [13, 14]. The “first epidemic” occurred in the 1940s and 1950s and principally affected premature infants in the USA and Western Europe. At that time unmonitored supplemental oxygen was the principal risk factor [15]. The “second epidemic” began in the 1970s, as a result of the increased survival rates of extremely premature infants in industrialized countries [16, 17]. Data from Canada, the USA and the UK show that the low birth weight and low gestational age of infants are risk factors for The “third epidemic” of ROP began in the 2000s due to the increased survival rates of premature infants in middle income countries including Eastern Europe, Latin America, India and China [18–22]. According to China’s population growth data [23], the number of newborns were extremely large in China before 1980. But the survival rate of premature infants is very low with the poor medical care. So the ROP incidence was low in that period. To 2000, China implemented the family planning policy to control the population. Although the level of neonatal care has improved significant, most families choose eugenics, and many premature babies were abandoned for treatment and rescue. The incidence of ROP was still low in that period. After 2000, China’s medical care has developed greatly, ROP screening got attention. The Chinese medical association formulated and improved the unified ROP screening guidelines twice in 2004 and 2014 [9]. According to the ROP screening guideline of China, the incidence of ROP in different regions of China is 6–18% [24, 25].

In this study, all subjects were severely premature infants who underwent ROP screening in the NICU of at Guangzhou First People’s Hospital from March 2013 to October 2017. There was a high incidence rate of ROP in multiple births, low birth weight, low gestational age, congenital dysplasia, developmental immaturity, sepsis, mechanical ventilation, and severe infection. The overall incidence of ROP was 31.65% (138 cases), and 13.99% (61 cases) of premature infants with ROP required treatment. These values are similar to those in many Chinese reports (from 10.8 to 17.6%) [26–28]; However, they are different from the values of Northwest China ROP report (5.5%) [29]. The incidence of ROP in China is lower than that in African countries, such as Kenya, Burundi, and Ethiopia [30]. The incidence of ROP in Kenya was 41.7, and 20.9% of premature infants with ROP required treatment [31]. However, the incidence of ROP in our study is higher than that in European countries, such as Switzerland and Sweden [32, 33]. The incidence of ROP in Switzerland was 9.3, and 1.2% of premature infants with ROP required treatment [32]. We assumed that the differences in ROP incidence were primarily related to the following factors: (1) some pregnant women did not receive regular antenatal care, and they’re maybe a significant error in the calculation of gestational age. (2) The survival rate of premature infants of very low birth weight and low gestational age was lower than that in developed countries. (3) The ROP screening guideline and the medical care of neonates in China were different to developed countries.

ROP is a disease involving multiple factors, and its exact etiology is not known. According to many reports, gestational age and birth weight are currently recognized as the primary risk factors for ROP [31]. Our results indicate that infants with a low birth weight or small gestational age or who require extended oxygen inhalation treatment have an increased incidence of ROP. This result is consistent with many other reports [34–36].

We also analyzed the correlation between ROP and individual factors in this study; the results showed that the factors significantly associated with the incidence of ROP include IVH, invasive examinations, and positive blood culture. However, the mechanisms by which these factors cause ROP are not clear. We speculate that these factors may lead to vital signs instability, and long-term, high-flow oxygen absorption is usually given as a treatment method.

In a logistic regression analysis, gestational age, birth weight and unmonitored supplemental oxygen were indicated as the primary risk factors for ROP [26]. Our study identified these three risk factors too. We also found two other independent risk factors, including minimum oxygen saturation, and average daily weight gain. We suspect that low oxygen saturation will prolong the duration of oxygen inhalation treatment, thereby increasing the incidence of ROP. The average daily weight gain is affected by a variety of disease factors such as growth factor secretion, which is associated with an increased incidence of ROP [37, 38].

With the rapid development of perinatal medicine and continuous improvements in treatment facilities, the survival rate for premature and low-birth weight infants has substantially improved, and the incidence of ROP has also significantly increased in China. Many studies have suggested that the up-regulated expression of VEGF plays an important role in the development of ROP [39], and anti-VEGF drugs can inhibit the over-expression of VEGF and thereby control intraocular angiogenesis and provide a good drug treatment for ROP [7, 40–42].

In report of the developed countries, the ROP that need treatment includes zone 1, any stage with plus disease; zone 1, stage 3, with or without plus disease; and zone 2, stage 2 or 3, with plus disease. The ROP that need watch and wait includes zone 1, stage 1 or 2 without plus disease and zone 2, stage 3 without plus disease. However, there is no uniform treatment threshold for ROP in China [9, 43]. Due to the special condition of China medical, many children will leave the NICU before the 54 weeks endpoint of the corrected gestational age. The ROP review should be performed at the outpatient clinic until the 54 weeks endpoint of the corrected gestational age. However, sometimes the ROP outpatient review cannot be carried out in time, furthermore some infants would lost control once leave hospital. In order to prevent the rapid progress of ROP, we would like to do retinal photocoagulation for some infants with ROP of zone3, stage3, and > 1 quadrant range. So there are little bit different in our report about the standard of treatment on ROP. In addition, the extremely severe premature infants born in our hospital, such as the extremely low born weight (< 1000 g), the extremely low gestational age (<26w) and systemic serious complications, would be transferred to the more specialized children’s hospital (Guangzhou Women and Children’s Medical Center) NICU. ROP incidence for those infants were not included in our study.

Retinal laser photocoagulation is a standard treatment for ROP. However, it can result in many problems, such as a wide range of retinal damage and the loss of vision in the photocoagulation area, and is not suitable for some patients, such as those with ROP in zone 1 or AP-ROP [44–46]. Anti-VEGF drugs may reduce complications when treating ROP. At the same time, anti-VEGF drugs can maintain the balance of VEGF and promote the continued development of retinal blood vessels in children with ROP [7, 40–42]. Many studies are currently ongoing to evaluate the use of anti-VEGF drugs in children with ROP [47, 48]. Anti-VEGF drugs for ROP treatment include Bevacizumab and Ranibizumab. Bevacizumab was used to treat ROP at first in 2006 [49, 50]. Bevacizumab is cheap and lasts long in ROP treatment, but it may have unknown systemic effects. Ranibizumab is a new anti-VEGF drug with a half-life of only 30 days and less systemic effects [51, 52]. In China, Bevacizumab couldn’t be used to treat ocular diseases, so we used Ranibizumab to treat ROP in this study.

In our study, children with ROP in zone 2 or 3 were treated with traditional retinal laser photocoagulation, whereas children with ROP in zone 1 and posterior polar invasion ROP were given Ranibizumab via intraocular injections. The recurrence of ROP in the laser treatment and anti-VEGF treatment groups were 42.86 and 41.67%, respectively. Because anti-VEGF drugs may induce systemic risks in premature infants, all infants who had ROP recurrence were retreated with laser photocoagulation.

The conditions of all children were controlled after retreatment was given until 54 weeks of PMA. No serious complications such as retinal detachment occurred in any patients in our study. We speculate that may be related to the fact that children with a very low birth weight (< 1000 g) and gestational age (< 26 weeks) were not included in this study. An analysis of the two groups showed that the gestational age and birth weight were significantly lower in the anti-VEGF treatment group than that in the laser treatment group. This result is similar to other reports [8, 44, 53–57].

In conclusion, in this study, we systemically analyzed the incidence of and risk factors for ROP in severely premature infants treated in the NICU at Guangzhou First People’s Hospital. The results of our study suggest that the incidence of ROP is 31.65% which will match those in middle-income countries, but higher than high-income countries. Anti-VEGF drugs could be preferred as a good treatment method for zone 1 ROP and aggressive posterior ROP.

## Abbreviations

anti-VEGF: Anti-vascular endothelial growth factor
AP-ROP: Aggressive posterior ROP
IVF: In vitro fertilization
IVH: Intravascular hemolysis
NICU: Neonatal intensive care unit
PDA: Patent ductus arteriosus
ROP: Retinopathy of prematurity
